# Variation in Pesticide Toxicity in the Western Honey Bee (*Apis mellifera*) Associated with Consuming Phytochemically Different Monofloral Honeys

**DOI:** 10.1007/s10886-024-01495-w

**Published:** 2024-05-18

**Authors:** Ling-Hsiu Liao, Wen-Yen Wu, May R. Berenbaum

**Affiliations:** Department of Entomology, University of Illinois Urbana-Champaign, Urbana, IL USA

**Keywords:** Honey bee, Phenolic acid, Flavonoids, Bifenthrin, LD_50_

## Abstract

Insecticide toxicity to insect herbivores has long been known to vary across different host plants; this phenomenon has been widely documented in both foliage-feeders and sap-feeders. Species-specific phytochemical content of hostplant tissues is assumed to determine the pattern of induction of insect enzymes that detoxify insecticides, but specific phytochemicals have rarely been linked to host plant-associated variation in pesticide toxicity. Moreover, no studies to date have examined the effects of nectar source identity and phytochemical composition on the toxicity of insecticides to pollinators. In this study, we compared LD_50_ values for the insecticide bifenthrin, a frequent contaminant of nectar and pollen in agroecosystems, in the western honey bee, *Apis mellifera*, consuming three phytochemically different monofloral honeys: *Nyssa ogeche* (tupelo), *Robinia pseudoacacia* (black locust), and *Fagopyrum esculentum* (buckwheat). We found that bifenthrin toxicity (LD_50_) values for honey bees across different honey diets is linked to their species-specific phytochemical content. The profiles of phenolic acids and flavonoids of buckwheat and locust honeys are richer than is the profile of tupelo honey, with buckwheat honey containing the highest total content of phytochemicals and associated with the highest bifenthrin LD_50_ in honey bees. The vector fitting in the ordination analysis revealed positive correlations between LD_50_ values and two honey phytochemical richness estimates, Chao1 and Abundance-based Coverage Estimator (ACE). These findings suggest unequal effects among different phytochemicals, consistent with the interpretation that certain compounds, including ones that are rare, may have a more pronounced effect in mitigating pesticide toxicity.

**Supplementary Information:**

The online version contains supplementary material available at 10.1007/s10886-024-01495-w.

## Introduction

Insecticide toxicity to insect herbivores has long been known to vary across different host plants. Among the mechanisms proposed by which host plant identity can influence the toxicity of an insecticide to an insect herbivore is that the species-specific phytochemical content of hostplant tissues determines the pattern of induction of detoxification enzymes, particularly the cytochrome P450 monooxygenases and carboxylesterases, capable of detoxifying the insecticide. This phenomenon was first described in interactions between polyphagous folivores and their host plants. Berry et al. (1980), e.g., showed that induction of aldrin epoxidase in *Peridroma saucia* (variegated cutworm) (Lepidoptera: Noctuidae) differed depending on hostplant identity and that this induction in turn affected the level of tolerance of three organophosphate insecticides.

Since then, host plant effects on insecticide toxicity have been demonstrated in other polyphagous (e.g., Karuppaiah et al. 2016; Saeed et al. 2019; Xue et al. 2010) as well as oligophagous (Prouty et al. 2021) lepidopterans. At least one coleopteran folivore, the oligophagous Colorado potato beetle, *Leptinotarsa decemlineata*, is known to experience differential toxicity in response to insecticide exposure depending on host plant identity (Ghidiu et al. 1990; Mahdavi et al. 1991). Mechanistically, differential activity of cytochrome P450 monooxygenases (P450s) can mediate the differential toxicity of insecticides to generalist lepidopterans. In the polyphagous tobacco cutworm, *Spodoptera litura*, with more than 120 recorded hostplant species, hostplant identity affected LD_50_ values for the organophosphate profenophos and the pyrethroid cypermethrin, with LD_50_ values for profenophos higher for larvae consuming castor than for larvae consuming soybean and the LD_50_ for cypermethrin lower for larvae consuming castor than for larvae consuming soybean. Activity levels of P450s were positively correlated with the LD_50_ of cypermethrin (Karuppaiah et al. 2016). Moreover, Guo et al. (2023) found that host-plant switching by the rice leaf-folder *Cnaophalocrocis medinalis* affected its susceptibility to abamectin and chlorpyrifos as well as activity of its detoxification enzymes [glutathione-*S*-transferases, “multifunctional oxidases” (including P450s) and carboxylesterases].

Although most extensively documented in folivores, hostplant identity can also influence the toxicity of insecticides to sap-sucking herbivores. Castle et al. (2008) demonstrated that the highly polyphagous silverleaf whitefly, *Bemisia tabaci*, recorded on more than 600 hostplant species, displayed higher LC_50_ values for a bifenthrin-endosulfan mixture when raised on broccoli or related cole crops than on cantaloupes or cotton. Similarly, Xie et al. (2011), comparing performance of *B. tabaci* across multiple host plants, demonstrated that all insecticides tested displayed lower toxicity on one host species, poinsettia (*Euphorbia pulcherrima*), relative to three other host plants, with the LC_50_ values for acetamiprid approximately 15-fold, tenfold, and 7.3-fold higher than for whiteflies on tomato, cucumber, and cabbage, respectively. In terms of mechanisms, Xie et al. (2011) linked induction effects of host plants to insecticide susceptibility in *B. tabaci*; glutathione-*S*-transferase (GST) and cytochrome P450 activity levels were lowest in the population on cucumber. Liang et al. (2007) linked the hostplant effects on insecticide toxicity to the induction of carboxyesterase activity in *Bemisia tabaci* biotype B and greenhouse whitefly, *Trialeurodes vaporariorum*. Njiru et al. (2023) found host plant modulation of acaricide resistance in the two-spottedspider mite, *Tetranychus urticae*, which use their mouthparts to pierce individual cells to remove their contents, to 13 acaricides with different modes of action and, with piperonyl butoxide synergism assays, demonstrated enhancement of toxicity of cyflumetofen in tomato but not bean, implicating P450s in detoxification. In addition, Dermauw et al. (2013) observed that when *T. urticae* mites were adapted from bean to a challenging host plant (tomato), their differentially expressed genes increased over generations, including P450 genes. Moreover, expression profiles of adapted mites resembled those of multipesticide-resistant strains, and this adaptation reduced their susceptibility to pesticides. This finding links host plant adaptation to pesticide resistance. 

Despite the broad recognition of impacts of hostplant identity on pesticide toxicity to foliage-feeding or piercing-sucking herbivores, there is virtually no literature on the effects of plant food source identity on insecticide toxicity to pollinators. As well, although enzymatic responses to host plant identity have been well-characterized, phytochemical traits of host plants that differentially affect pesticide toxicity have almost never been documented. As a consumer of plant nectar, pollen and their processed forms honey and beebread, the western honey bee, *Apis mellifera*, in particular should be susceptible to effects, positive and negative, of phytochemicals due to the considerable diversity of nectar sources exploited by this highly polylectic species and to its ability to concentrate and convert nectar into honey as a storable food resource. Some nectars, e.g., contain phytochemicals toxic to bees; conversion of these nectars into honey can lead to hive collapse (Bischoff and Moiseff 2023). Others contain phytochemicals that can be beneficial, providing antioxidant and antimicrobial activities (Berenbaum and Calla 2021). Most importantly, certain phytochemicals found in honey can ameliorate pesticide toxicity in bees by upregulating cytochrome P450 enzymes (Mao et al. 2011, 2013).

In this study, we set out to determine whether pollinators, like other herbivores, experience differential toxicity of insecticides depending on the species identity of the nectar sources used to make honey. *Apis mellifera*, the western honey bee, was selected for this study as a test case due to its ability to collect and process into honey nectars from many different plant species, encountering a broad range of nectar phytochemicals in the process. As well, because bees concentrate nectar in converting it to honey, effects of nectar source identity on pesticide toxicity should be more likely to be detectable, particularly if induction of detoxification enzymes is dose-dependent. To date, individual honey constituents have been tested for their effects on insecticide toxicity (Arathi and Bernklau 2021; Liao et al. 2020, 2017; Mao et al. 2011, 2013; Mitton et al. 2020; Wong et al. 2018), but honey bees encounter phytochemicals in complex mixtures, not in isolation, when they eat honey, and there are few if any studies of the effects of the phytochemical composition of honey on insecticide tolerance. Accordingly, we tested the toxicity of a pyrethroid insecticide, bifenthrin, on adult honey bees consuming three types of monofloral honeys–i.e., honeys that derive 50% or more of their constituent nectar from a single nectar source. Bifenthrin is both highly toxic to honey bees (USEPA OPP Pesticide Ecotoxicity Database) and frequently encountered in agroecosystems in which bees forage and in the hive environment; accordingly, we selected it to serve as a representative pesticide to determine effects of honey identity on pesticide toxicity. In a recent study of pesticide residues in bee-attractive border plantings, e.g., bifenthrin was the most frequently detected among 33 pesticides, found in 44 percent of all samples (Ward et al. 2022). As well, in a four-year monitoring survey of honey bee exposure to pesticide residues in hives in China’s main honey-producing areas, bifenthrin had the third-highest detection rate, 19.7% (Xiao et al. 2022), behind only the fungicide carbendazim, with a detection rate of 45%, and the in-hive acaricide tau-fluvalinate, with a detection rate of 36.8%. In addition, we analyzed the correlations between alpha diversity metrics, which measure the richness and evenness of the “community” of phytochemicals contained in honey, and the LD_50_ values of bees consuming a pesticide administered in five honey diets. This analysis allowed us to measure correlations between the identity and diversity of honey phytochemicals consumed and observed toxic effects of pesticide exposure.

## Methods

### Identification and Quantification of Phenolic Components of Honeys

Monofloral honeys from each of three plant families known to differ in phytochemical content and composition (Gheldof et al. 2002) were selected for this study: white tupelo (Nyssaceae: *Nyssa ogeche*; commercial tupelo honey from Wewahitchka, FL, USA), black locust (Fabaceae: *Robinia pseudoacacia*; commercial locust blossom raw honey, from Plains, PA, USA), and buckwheat (Polygonaceae: *Fagopyrum esculentum*; commercial buckwheat honey, from Plains, PA, USA). Methods for honey sample preparation and high-pressure liquid chromatography (HPLC) analysis were adapted from those reported by Gheldof et al. (2002) and Michalkiewicz et al. (2008). Twenty grams of tupelo or locust honey or 10 g of buckwheat honey were dissolved in 100 mL of acidified deionized water (pH 2.0) and filtered through solid-phase extraction (SPE) cartridges (186008718, Waters Corporation, Milford, MA) on a vacuum station at flow rate < 5 mL/min. The loading quantity of buckwheat honey was halved to avoid saturating and blocking SPE cartridges. After washing each cartridge with an additional 100 mL acidified water to remove sugars and polar compounds, 50 mL methanol were eluted to recover the adsorbed phenolic acids and flavonoids. The methanol extract was concentrated using a rotary evaporator at 30°C and the solid extracts were then redissolved in 1 mL (tupelo and locust) or 0.5 mL (buckwheat) methanol containing methyl 4-hydroxybenzoate (200 μg/mL) as internal standard. The supernatant of reconstituted extract, centrifuged at 18,000 g RCF for 30 s, was used for HPLC analysis.

HPLC analysis was performed on a Phenomenex® Gemini C18 column (150 mm by 2 mm, 5 μm) with a Shimadzu Prominence SPD-M20A photodiode array detector (PDA; scanning range: 190–450 nm, slit of 1.2 nm, acquisition rate of 1.5625 Hz, and flow in the cell temperature of 40 °C). The column oven temperature was maintained at 40°C as well. Gradient elution and variable total flow rate of the mobile phase were carried out for obtaining an optimized chromatographic peak separation and for keeping the operating pressure below the upper limit of the pump and system. The mobile phase consisted of 0.5% formic acid in water (phase A) and methanol (phase B). Before the sample injection, the mobile phase was kept at 20% B for 15 min at 0.2 mL/min flow rate. After the injection (0 min), the mobile phase was delivered in linear gradient mode as follows: in 0.01 min decreasing 15% B, 0.01–5 min 15% B, 9–16 min 25% B, 30–34 min 45% B, 44 min 48% B, 50–65 min 60% B, 66–71 min 95% B, and holding for 4 min. The flow rate was also changed linearly after sample injection, decreasing from 0.2 to 0.1 mL/min over four min, maintained from 4–7 min at 0.1 mL/min, from 9–16 min at 0.15 mL/min, from 17–24 min at 0.18 mL/min, from 28–38 min at 0.16 mL/min, for 39 min at 0.18 mL/min, from 43–71 min at 0.2 mL/min; and holding for 4 min.

Components were identified and quantified by comparing with reference standard retention time, absorbance spectral characteristics, and integrated area of absorbance peaks detected at their best detection wavelength (Table S1). The quantification was calibrated via normalization of the peak areas by referring to the internal standard and calibration curves established with known concentrations of standard chemicals.

### Effects of Honey Phytochemicals on Acute Pesticide Toxicity

For bioassays assessing pesticide toxicity to bees on different monofloral honey diets, the method of Wong et al (2018) was used to evaluate the impact of consuming three different monofloral honeys on bifenthrin median lethal dose (LD_50_) values. Honey bees were obtained from apiaries of the University of Illinois Bee Research Facility located in Urbana, Champaign County, IL (40°07′52"N 88°08′43"W and 40°07′38"N 88°10′31"W) in summer 2018. Frames of capped brood were collected from three naturally mated queen colonies and then incubated in a dark room at 34°C to obtain newly emerged worker bees. The day-old bees, collected within 24 h of eclosion, were introduced into cages in groups of 10 individuals (except for two cages, which inadvertently contained 11 bees). Each cage, following methods used in earlier studies (Liao et al. 2020, 2017, 2019), was equipped with four 2-mL microcentrifuge tube feeders; three feeders provided a formulated honey diet and one provided water. The experiment comprised five diet treatments: tupelo, locust, and buckwheat honey in separate cages, a choice treatment (TLB-Choice) offering three honey options, and a sugar control that represents the average sugar proportions in the honeys (40% fructose: 29% glucose: 1% sucrose) as documented in previous studies (Gardiner 2015; Pasini et al. 2013; White and Doner 1980). All diets contained casein (C3400, Sigma–Aldrich Co. LLC., St. Louis, MO) at a ratio of 1:12 protein to carbohydrate as a phytochemical-free protein source. Three days after caging, surviving bees (9–11 bees per cage) within their cages were chilled with ice to keep them immobilized and were then individually treated topically with bifenthrin in acetone or acetone alone as a solvent control. We evaluated the effects of honey on the bifenthrin (LD_50_) with 1 µl acetone containing concentrations of bifenthrin encompassing 0 ppb, 120 ppb, 150 ppb, 240 ppb, 300 ppb, 600 ppb, 1200 ppb, 1500 ppb, 2400 ppb, and 3000 ppb. Three to nine replicates of each concentration in each treatment were tested from each of three naturally mated queen colonies, except for 120 ppb, which had two replicates for two colonies, for a total of 5159 bees. All three hives and cage replicates were carried out within a 24-day period.

Probit analysis was conducted to estimate LD_50_ values using IBM SPSS Statistics (version 24, SPSS Inc., Chicago, IL, USA). A heterogeneity factor was included in the calculation of 95% confidence limits if the significance level of Pearson Goodness-of-Fit Test was below 0.15 (Norušis 2007). Significant differences between LD_50_ values were determined by estimation of confidence intervals of the relative median potency (RMP) when values of the 95% confidence interval of relative median potency did not include “1”.

### Analysis of the Phytochemical Composition of Honeys and their Associations with Honey Bees

In phytochemical studies, alpha diversity indices have been used to assess of phytochemical diversity to provide a quantitative measure of the composition of naturally occurring mixtures (Hilker 2014; Wetzel and Whitehead 2020). The indices have facilitated comparisons of phytochemical diversity among host samples and have been used as quantitative indices to develop models to study the effects of phytochemical diversity on herbivore performance (Glassmire et al. 2020), ecological interactions (Cacho et al. 2015; Doyle 2009; Richards et al. 2015), and evolutionary processes (Morris et al. 2014; Tewes et al. 2018). We used several common diversity indices for a comprehensive characterization of phytochemical richness and evenness, including Richness, Shannon–Wiener diversity Index (Shannon), Inverse Simpson diversity Index (inv_ Simpson), Pielou's Evenness Index (Pielou), and extrapolated richness estimators (Chao et al. 2014), including the Chao1 richness estimator (Chao1) and the Abundance-based Coverage Estimator (ACE), using the 'vegan' package (Dixon 2003; Oksanen et al. 2022) in R (R Core Team 2023). The Richness index quantified the total number of phytochemicals in each honey sample; the Shannon index and the inverse Simpson index were used to measure both richness and evenness; the Pielou index measured the evenness of the compound distribution; and Chao1 and ACE estimated the total number of phytochemicals, considering both detected and undetected ones.

To assess the differences in phytochemical diversity among the three honey samples, we first used Levene's test for equality of variances to evaluate the homogeneity of variances. If homogeneity of variances was confirmed (Levene's test, p > 0.05), we used analysis of variance (ANOVA) followed by Scheffé's post-hoc analysis. If homogeneity of variances was violated, indicating unequal variances, we performed the Kruskal–Wallis rank test with Dunn's test for pairwise comparisons. A significance level of α = 0.05 was used in the tests. For the analysis of the phytochemical composition of honey and its effects on pesticide toxicity, we employed multivariate analysis with non-metric multidimensional scaling (NMDS) and the envfit function from the R package 'vegan' (Dixon 2003; Oksanen et al. 2022). Prior to analysis, the phytochemical units in honey were converted to μM to assess the bioavailable concentrations of the phytochemicals in the honey diet. NMDS plots, in conjunction with a stress value and the Adonis index, were utilized to evaluate the clustering of honey samples based on phytochemistry (Bray–Curtis distance, k = 5). A stress value close to 0 indicated a good fit to the NMDS plot, while the Adonis test provided R-square and p values to assess the significance of the observed group differences. Additionally, the envfit function for multiple regression with 999 permutations was used to fit variables (vectors) to the NMDS ordination, regardless of whether explained variables were part of the original analysis that generated the plot. This function facilitated the visualization and quantification of relationships between variables by aligning environmental factors with the ordination plot (Dixon 2003). This approach revealed associations of the variables with the phytochemicals present in the honey samples by correlating them with the underlying ordination axes. It also helped to characterize relationships between variables; for example, angles between vectors (variables) on the NMDS ordination plot indicate their correlations (Šmilauer and Lepš 2014). These variables included individual phytochemicals, alpha diversity metrics of honey phytochemical composition, and average 24-h LD_50_ values to represent toxicity of pesticides to bees on different honey diets.

## Results

### Monofloral Honey Characteristics: Phytochemicals and Alpha-Diversity

The major phytochemical constituents of the three monofloral honey are presented in Table 1. Buckwheat honey is characterized by its richness in phenolic acids, especially *p*-hydroxybenzoic acid, and surpassed tupelo and locust honey in its levels of pinobanksin and pinocembrin (Table 1). Tupelo honey contained high levels of the sesquiterpene abscisic acid and greater concentrations of quercetin and kaempferol, while locust honey was characterized by its higher hyperoside content.

**Table 1:**
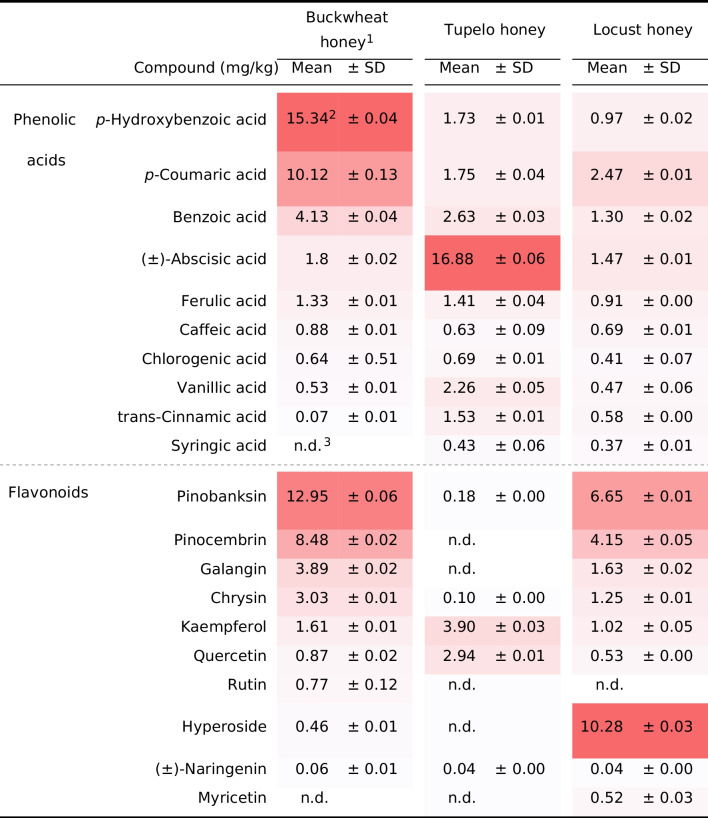
Major phytochemical constituents of three monofloral honeys

^1^ *n* = 6 in each monofloral honey

^2^ Colors indicator ranges from white (n.d.) to red (highest) in each monofloral honey

^3^ n.d. = not detected

As reflected by diversity indices, buckwheat and locust honeys exhibited a phytochemical profile with higher richness than tupelo honey. Buckwheat honey had the highest Chao1 estimation at 21.89 ± 1.46 of total phytochemicals, followed by locust honey at 19.38 ± 0.20, and tupelo honey at 16.50 ± 0.13. Both buckwheat and locust honeys had significantly higher estimation of phytochemicals than does tupelo honey (Table 2 and S2) (p < 0.05, Dunn's test after Kruskal–Wallis rank test). In the ACE estimation of honey phytochemicals, a similar pattern was observed, with buckwheat honey having the highest value at 20.51 ± 0.48, followed by locust honey at 19.68 ± 0.20, and tupelo honey at 18.47 ± 0.17. Both buckwheat and locust honeys showed significantly higher values than tupelo honey (p < 0.05, Scheffé post hoc test after ANOVA), with no significant difference between buckwheat and locust honeys. The average phytochemical richness values were 19.33, 18, and 15.17 for locust, buckwheat, and tupelo honeys, respectively, with locust honey having a significantly higher richness value than tupelo honey (p < 0.05, Dunn’s test after Kruskal–Wallis rank test), while no significant difference was observed between locust and buckwheat honey. However, with respect to phytochemical evenness, locust honey had the highest evenness (Pielou index: 0.60); Pielou evenness values for buckwheat (0.50) and tupelo honey (0.53) were lower. In addition, locust honey was characterized by the highest values for the Shannon and Inverse Simpson indices.

**Table 2:**
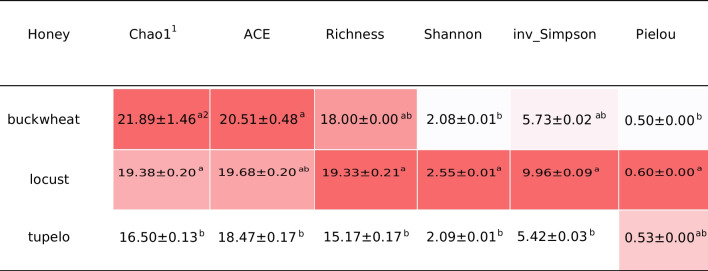
Alpha diversity metrics of honey phytochemical compositions (mean ± se)

^1^Chao1: Chao1 richness estimator; ACE: Abundance-based Coverage Estimator; Shannon: Shannon–Wiener diversity Index; inv_Simpson: Inverse Simpson diversity Index; Pielou: Pielou's Evenness Index

^2^Superscripted lowercase letters following alpha diversity metrics indicate statistical differences in phytochemical composition of honeys in **µ**M (p < 0.05, n = 18, df = 2, 15, Scheffé post hoc test after ANOVA or Dunn’s test after Kruskal–Wallis rank test); Color indicator ranges from white (lowest) to red (highest) for the mean values of each diversity metric

### Effects of Honey on Honey Bees: Pesticide Acute Toxicity

In terms of acute toxicity, the median lethal dose (LD_50_) values for bifenthrin in bees on each of the four honey-containing diets were higher than those for bees on the phytochemical-free diet at both 24 h and 48 h (Table 3). The LD_50_ values for bifenthrin for bees on the buckwheat honey diet were greater than the LD_50_ values for bees on the phytochemical-free sugar diet [the relative median potency (RMP) = 0.73 (0.61–0.87, 95% CI) at 24 h and RMP = 0.77 (0.63–0.93, 95% CI) at 48 h]. Bees on the TLB-Choice diet also had greater LD_50_ values at 24 h than the bees on the sugar diet [RMP = 0.80 (0.67–0.95, 95% CI)].

**Table 3:**
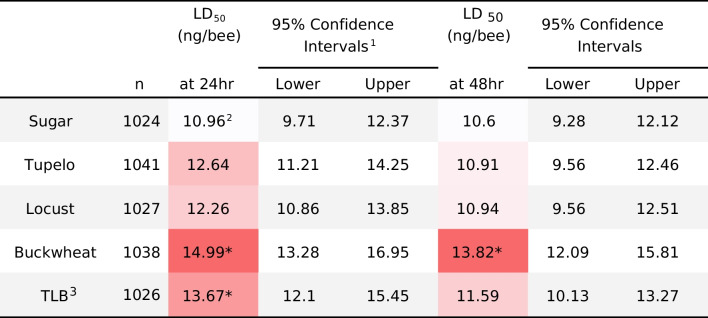
Median lethal dose (LD_50_) of bifenthrin to adult honey bees on three different monofloral honey diets and the sugar control diet

^1^ a heterogeneity factor is used in the calculation of confidence limits, logarithm base = 10, due to Pearson Goodness-of-Fit Test p < 0.15

^2^ Color indicator ranges from white (lowest) to red (highest) for median lethal dose levels

^3^ TLB: free choice among three honeys

^*^Significantly different from the sugar control, based on relative median potency (RMP) analysis

### Honey Phytochemical Composition and Associations with Honey Bees

**Fig. 1:**
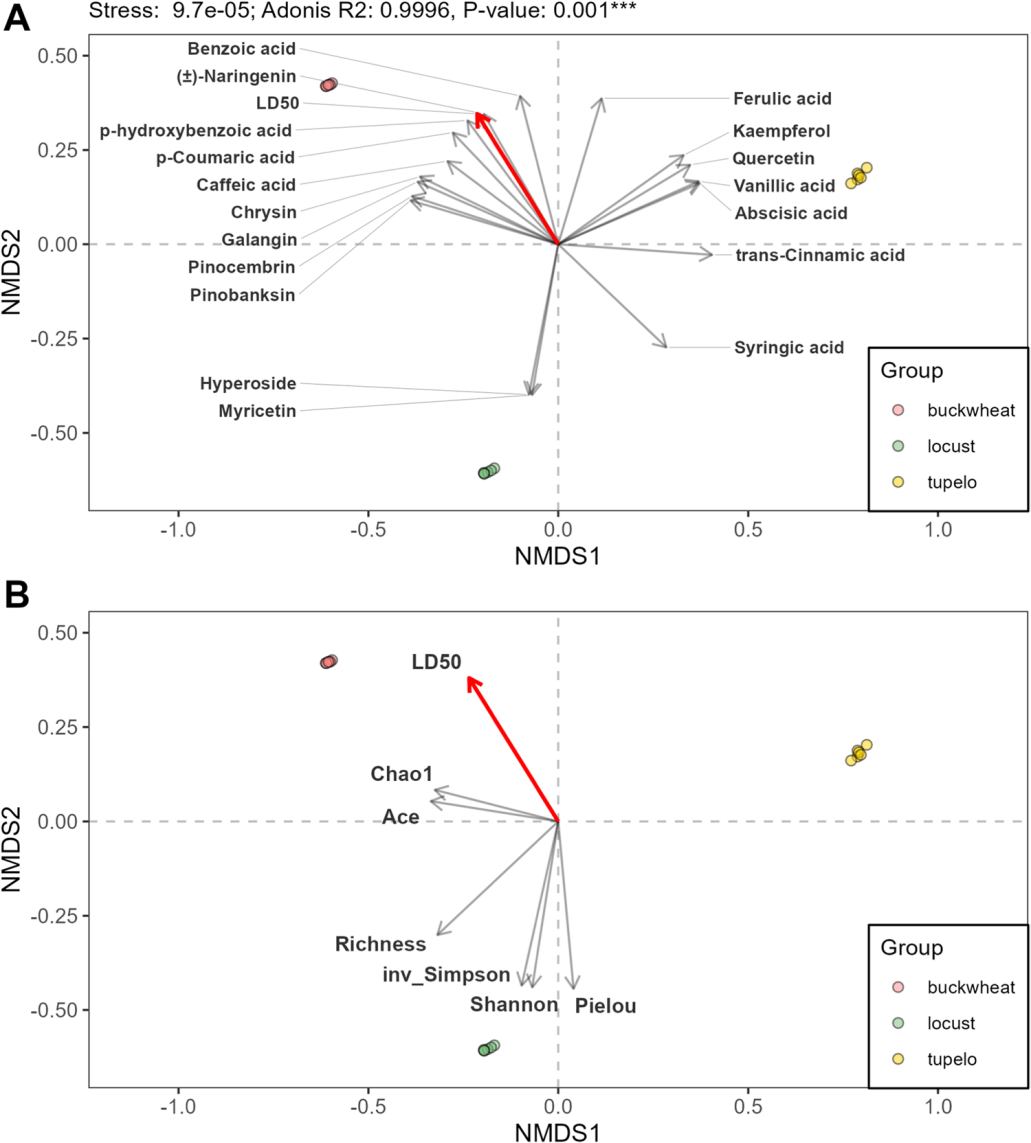
The nonmetric multidimensional scaling (NMDS) plot illustrates the phytochemical distributions among samples from three monofloral honey, based on the Bray–Curtis distance (n = 18; Adonis: 0.99, p < 0.001). The NMDS plot also displays vectors for phytochemicals (**A**), alpha diversity metrics of honey phytochemical composition (**B**), and LD_50_ of bifenthrin for honey bees (red), as determined by the envfit function. The direction of the vector arrows indicates the maximum gradient direction of the variable (the direction of the most rapid change in the variable), and the arrow length is proportional to the squared correlation coefficient with honey samples. The angle between two vectors indicates the direction of the relationship between them, with an acute angle indicating a positive correlation, a perpendicular angle indicating an uncorrelated relationship, and obtuse angles indicating a negative correlation. Only variables that are statistically significant are shown (p < 0.05; based on 999 permutations; Table S3)

The non-metric multidimensional scaling (NMDS) plot (Fig. 1), based on the Bray–Curtis distance, illustrated the grouping of honey samples according to their phytochemicals (Adonis: 0.99, p < 0.001). With the exception of rutin and chlorogenic acid, phytochemicals showed statistically significant associations with honey types (p < 0.01; based on 999 permutations, Fig. 1A; Table S3). Similarly, LD_50_ values and alpha diversity metrics of phytochemicals showed statistically significant associations with honey phytochemical composition (Fig. 1B; Table S3).

In addition, our analysis using envfit evaluated the relationships between honey bee LD_50_ and phytochemical variables within the NMDS ordination plots (Fig. 1), suggesting that certain phytochemicals influence susceptibility to bifenthrin pesticide toxicity more than others. For example, some phytochemical variables, such as *p*-hydroxybenzoic acid, *p*-coumaric acid, benzoic acid, caffeic acid, naringenin, pinobanksin, pinocembrin, galangin, and chrysin, pointed in a similar direction to the LD_50_ variable, suggesting positive correlations, while others showed negative correlations (hyperoside, myricetin, syringic acid and trans-cinnamic acid) or were uncorrelated (Fig. 1).

Because honey bees are exposed to phytochemicals in mixtures rather than in isolation, our analysis examined the correlations between honey bee LD_50_ and alpha-diversity metrics of phytochemicals in honey. Acute angles reflected the relationship between honey bee LD_50_ and the Chao1 richness estimate (Chao1), as well as between LD_50_ and the Abundance-based Coverage Estimator (ACE), suggesting a positive correlation (Fig. 1B). This finding indicates that, as the richness estimate increases, so does the LD_50_ value. Both Chao1 and ACE estimate phytochemical richness by weighting more heavily compounds present in low abundance to account for compounds that may have been missed during the analysis (Chao et al. 2014; Xia and Sun 2023). By contrast, the observed nearly perpendicular angles between LD_50_ and the richness index indicate a lack of correlation. However, when alpha diversity metrics that incorporate evenness, such as Pielou's Evenness Index, Shannon's Index, and inverse Simpson's Index, are considered, we observed inverted angles. This finding indicates a negative correlation between honey bee LD_50_ and these metrics, suggesting that, as the evenness of phytochemical distribution increases, the LD_50_ tends to decrease.

## Discussion

In our study, the significant associations between LD_50_ with honey phytochemical composition suggest multiple phytochemicals of honey influence susceptibility to bifenthrin. Although in honeys some properties are associated with sugars (Berenbaum and Calla 2021), in most honeys biological activity results from their phytochemical profile, which varies substantially according to availability of floral sources for foragers. Examining only three monofloral honeys to evaluate functional differences among honeys varying in phytochemical diversity is a limitation of our study in that it captures only a minuscule sample of honey phytochemical diversity. As well, accurately estimating phytochemical ingestion by bees is challenging. The TLB-Choice diet, designed to allow bees to choose among the three honeys to simulate behavioral regulation of phytochemical ingestion, did not allow us to determine the exact amounts of each honey type consumed and thus to estimate the diversity of phytochemicals ingested by the bees choosing their food. Notwithstanding these limitations, we were able to document differences in biological activities of these three honeys that are directly relevant to bee health—that is, sensitivity to a pesticide of agricultural importance.

A well-documented property of honey relevant to bee health is its ability to up-regulate specific detoxification enzymes (Johnson et al. 2012; Mao et al. 2013). Such activity is reflected in the diet-dependent differences in bifenthrin LD_50_ we observed; relative to the sugar diet, the median lethal dose of bifenthrin increased with consumption of honey with greater diversity of phytochemicals (Chao1 richness estimate and Abundance-based Coverage Estimator (ACE)). This finding is consistent with increased pesticide detoxification after ingestion of individual phytochemicals found in honey. Multiple studies have demonstrated amelioration of pesticide toxicity by consumption of certain phytochemicals individually (Arathi and Bernklau 2021; Liao et al. 2020, 2017; Mao et al. 2013; Mitton et al. 2020; Wong et al. 2018). Along the same lines, Ardalani et al. (2021a) demonstrated that bees consuming quercetin displayed reduced residual concentrations of ingested imidacloprid. To date, however, Ardanali et al. (2021b) is the only study of impacts of diets containing natural mixtures of phytochemicals on pesticide metabolism; these authors reported that flavonoids in nectar and pollen diets reduce the residual concentrations of imidacloprid and tau-fluvalinate.

Fully characterizing the beneficial non-nutritive effects of honey phytochemicals will require a multifactorial approach. The main mechanism for increased pesticide detoxification by complex mixtures of phytochemicals in honey relative to a phytochemical-free sugar diet is ostensibly the collective induction of detoxification pathways, particularly CYP6AS and CYP9Q subfamilies (Haas et al. 2022a; Mao et al. 2009). Induction of cytochrome P450s occurs in honey bees consuming individual phytochemicals, including *p*-coumaric acid, pinocembrin, pinobanksin and pinobanksin 5-methyl ether (Mao et al. 2013); of these, present in all three honeys were *p*-coumaric acid and pinobanksin, albeit in different concentrations. CYP6AS subfamily enzymes and CYP9Q3 metabolize quercetin and are induced by *p*-coumaric acid (Mao et al. 2009, 2013, 2015); phytochemical-rich honeys induced four CYP6AS transcripts and CYP9Q3 transcripts, which likely also increased the overall capacity for detoxification of natural and synthetic xenobiotics (Liao et al., in preparation). Additionally, CYP9Q3 is involved in the detoxification of multiple insecticides, including the pyrethroid tau-fluvalinate and the organophosphate coumaphos (Mao et al. 2011), the N-cyanoamidine neonicotinoid thiacloprid (Manjon et al. 2018), the butenolide flupyradifurone (Belden 2022), and the anthranilic diamide chlorantraniliprole (Haas et al. 2022a). A phylogenomic analysis showed that functional CYP9Q orthologs are generally conserved across bee families (Haas et al. 2022b), suggesting their importance in the adaptation of bees to environmental stress. Rather than consuming phytochemicals individually, however, honey bees ingest mixtures of phytochemicals while feeding on honey. Herbivore-plant ecological interactions correlate with mixtures of host phytochemicals (Marion et al. 2015; Petrén et al. 2023), suggesting that studies of the effects of phytochemicals of honey on bees should take into account the overall diversity of honey phytochemicals. Our multivariate analysis revealed the relationship between bifenthrin toxicity and the diversity of phytochemicals of the three honeys, reflected by positive correlations between LD_50_ and richness estimates (Chao1 and ACE), indicating higher LD_50_ with increased richness, and negative correlations with alpha diversity metrics incorporating evenness of phytochemical composition. These results suggest that certain specific compounds may have a more pronounced effect than others in reducing pesticide toxicity. In addition, the positive correlation observed between richness estimates (Chao1 and ACE) and LD_50_ suggests that low-abundance phytochemicals, which are weighted more heavily in these estimates (Chao et al. 2014; Xia and Sun 2023), may also contribute to the reduction of bifenthrin pesticide toxicity.

## Conclusions

Although up-regulation of xenobiotic detoxification pathways in honey bees in response to honey likely evolved in response to potentially toxic phytochemicals, induction of detoxification pathways by phytochemical-rich honeys is likely beneficial in contemporary pesticide-contaminated environments. Impacts of reduced phytochemical diversity in the diet provide insights into the consequences of reduced floral resource diversity and intensively farmed agroecosystems (Decourtye et al. 2010). It is important to note, however, that phytochemicals that are not derived from floral nectars are also found in honey (Nešović et al. 2020). As Soler et al. (1995) point out, honeys contain not only phytochemicals derived from nectar but also “the characteristic flavonoids from propolis and/or beeswax (chrysin, galangin, tectochrysin, pinocembrin and pinobanksin)”, which in our study have a positive correlation with the LD_50_ variable. Truchado et al. (2008) specifically point out that the flavonoid aglycones in acacia, or locust, honey (*R. pseudoacacia*) derive from propolis, the substance made by bees from resins collected from plants that are mixed with wax and saliva. The phenolic acid *p*-coumaric acid is a frequent component of European propolis (Hegazi et al. 2000). Propolis-derived flavonoids, including pinocembrin, pinobanksin, and galangin, are absent or present in very low concentrations in our tupelo honey samples relative to the amounts in locust and buckwheat honey. Buckwheat honey in particular is rich in these flavonoids. Mao et al. (2013) reported that *p*-coumaric acid is the strongest inducer of the detoxification enzyme CYP9Q3 among phenolic acids, and, among flavonoids, chrysin and naringenin were more effective at inducing CYP9Q3 than were pinocembrin and galangin; pinobanksin 5-methyl ether is “highly effective.” Thus, for bees, plant diversity of landscapes other than that representing nectar sources may have hitherto unrecognized or underestimated health benefits in terms of pesticide toxicity challenges.

In conclusion, honey, the principal stored food product during a substantial proportion of the lifecycle of the honey bee, likely has greater importance in honey bee health than previously recognized, particularly if bees can self-regulate induction of detoxification enzymes as they apparently self-medicate in the presence of pathogens (Gherman et al. 2014; Spivak et al. 2019; Tihelka 2018). Variation in honey phytochemical content may help equip bees with defenses against both natural and synthetic xenobiotics. Potential applications arising from our findings may include landscape diversification plans aimed at optimizing the phytochemical content of non-crop flora to increase the likelihood of occurrence of honey phytochemicals, particularly those introduced into the hive via resin-collecting and propolis production, that can upregulate detoxification enzymes, to promote year-round good health.

## Supplementary Information

Below is the link to the electronic supplementary material.

Supplementary file1 (DOCX 30.4 KB)

## Data Availability

The datasets generated and analyzed in this study are available on the Illinois Data Bank (10.13012/B2IDB-6733018_V1) (Liao et al. 2024).
