# Supplementary material for: Variation in Pesticide Toxicity in the Western Honey Bee (Apis mellifera) Associated with Consuming Phytochemically Different Monofloral Honeys
Source: J Chem Ecol. 2024 May 18;50(7-8):397–408. doi: 10.1007/s10886-024-01495-w (PMC11399171; doi:10.1007/s10886-024-01495-w)
Supplement: Supplementary file 1 — Supplementary file1 (DOCX 30.4 KB) [file 10886_2024_1495_MOESM1_ESM.docx]

**Table S1**. Sources of reference standards used in the HPLC analysis of phytochemical profiles of three monofloral honeys

| Chemical | Retention time (minutes) | Detection λ (nm) | LOD ^1^ (mg/kg) | Brand | Catalog # | CAS # |
| --- | --- | --- | --- | --- | --- | --- |
| *p*-Hydroxybenzoic acid | 15.231 | 254 | 0.270 | Supelco | H5376 | 99-96-7 |
| Chlorogenic acid | 16.581 | 340 | 0.053 | Sigma-Aldrich | C3878 | 327-97-9 |
| Vanillic acid | 17.401 | 254 | 0.097 | Sigma-Aldrich | 94770 | 121-34-6 |
| Caffeic acid | 18.353 | 285 | 0.052 | Sigma-Aldrich | C0625 | 331-39-5 |
| Syringic acid | 19.207 | 285 | 0.035 | Cayman chemical | 19196 | 530-57-4 |
| *p*-Coumaric acid | 24.973 | 285 | 0.039 | Sigma-Aldrich | C9008 | 501-98-4 |
| Ferulic acid | 26.972 | 285 | 0.024 | Sigma-Aldrich | 128708 | 537-98-4 |
| Benzoic acid | 28.061 | 254 | 0.013 | Sigma-Aldrich | B3250 | 65-85-0 |
| Hyperoside | 34.388 | 254 | 0.067 | Sigma-Aldrich | 00180585 | 482-36-0 |
| Rutin | 34.701 | 254 | 0.166 | Thermo Scientific | 132390050 | 153-18-4 |
| (±)-Abscisic acid | 36.638 | 254 | 0.033 | Sigma-Aldrich | 862169 | 14375-45-2 |
| Myricetin | 37.557 | 254 | 0.198 | Sigma-Aldrich | M6760 | 529-44-2 |
| trans-Cinnamic acid | 38.416 | 285 | 0.007 | Sigma-Aldrich | C80857 | 140-10-3 |
| pinobanksin | 42.645 | 285 | 0.021 | Sigma-Aldrich | 68530 | 548-82-3 |
| (±)-Naringenin | 43.643 | 285 | 0.006 | Sigma-Aldrich | N5893 | 67604-48-2 |
| Quercetin | 44.925 | 254 | 0.167 | Sigma-Aldrich | Q4951 | 117-39-5 |
| Kaempferol | 52.248 | 254 | 0.179 | Cayman chemical | 11852 | 520-18-3 |
| Pinocembrin | 55.719 | 285 | 0.090 | Sigma-Aldrich | P5239 | 480-39-7 |
| Chrysin | 59.377 | 254 | 0.097 | Sigma-Aldrich | C80105 | 480-40-0 |
| Galangin | 62.011 | 254 | 0.032 | Cayman chemical | 15948 | 548-83-4 |
| Methyl 4-hydroxybenzoate  (Internal standard) | 29.299 | 254 |  | Sigma-Aldrich | H3647 | 99-96-7 |

^1^ LOD: limit of detection; determined according to the method by Allegrini and Olivieri (2020). The reported LODs are one-twentieth of the analytical instrument's limit of detection, considering the 20-fold concentrated extraction of phytochemicals from honey samples.

**Table S2.** Alpha diversity based on phytochemical composition (in µM) of each honey sample

| Sample | Chao1 | ACE | Richness | Shannon | inv_Simpson | Pielou |
| --- | --- | --- | --- | --- | --- | --- |
| BW1 | 21.00 | 20.844 | 18 | 2.071 | 5.713 | 0.497 |
| BW2 | 18.33 | 18.816 | 18 | 2.099 | 5.819 | 0.503 |
| BW3 | 24.00 | 20.488 | 18 | 2.085 | 5.759 | 0.500 |
| BW4 | 28.00 | 22.328 | 18 | 2.064 | 5.688 | 0.495 |
| BW5 | 19.00 | 19.743 | 18 | 2.073 | 5.720 | 0.497 |
| BW6 | 21.00 | 20.844 | 18 | 2.059 | 5.664 | 0.494 |
| Tup1 | 16.50 | 18.209 | 15 | 2.088 | 5.425 | 0.534 |
| Tup2 | 16.00 | 18.812 | 15 | 2.062 | 5.311 | 0.528 |
| Tup3 | 16.50 | 18.209 | 15 | 2.085 | 5.421 | 0.534 |
| Tup4 | 17.00 | 19.188 | 16 | 2.113 | 5.514 | 0.528 |
| Tup5 | 16.50 | 18.209 | 15 | 2.086 | 5.418 | 0.534 |
| Tup6 | 16.50 | 18.209 | 15 | 2.088 | 5.415 | 0.534 |
| Loc1 | 20.00 | 20.285 | 20 | 2.588 | 10.274 | 0.599 |
| Loc2 | 19.00 | 19.291 | 19 | 2.532 | 9.792 | 0.596 |
| Loc3 | 19.00 | 19.281 | 19 | 2.538 | 9.862 | 0.597 |
| Loc4 | 19.00 | 19.291 | 19 | 2.537 | 9.866 | 0.597 |
| Loc5 | 20.00 | 20.272 | 20 | 2.581 | 10.186 | 0.597 |
| Loc6 | 19.25 | 19.687 | 19 | 2.529 | 9.798 | 0.595 |
| post hoc test^1^ | Duun | Scheffé | Duun | Scheffé | Duun | Duun |

^1^ Scheffé: Scheffé post hoc test was used after ANOVA; Duun: Dunn's post hoc test was used after Kruskal-Wallis rank test.

**Table S3.** Correlations of variables with NMDS ordinations of phytochemical compositions of honey samples using the envfit function in the vegan package

|  | NMDS1 | NMDS2 | r^2^ | Pr(>r) |  |
| --- | --- | --- | --- | --- | --- |
| *p*-Hydroxybenzoic acid | -0.589 | 0.808 | 0.9999 | 0.001 | *** |
| *p*-Coumaric acid | -0.685 | 0.729 | 0.9995 | 0.001 | *** |
| Benzoic acid | -0.248 | 0.969 | 0.9990 | 0.001 | *** |
| Abscisic acid | 0.917 | 0.398 | 0.9994 | 0.001 | *** |
| Ferulic acid | 0.283 | 0.959 | 0.9857 | 0.001 | *** |
| Caffeic acid | -0.799 | 0.602 | 0.8112 | 0.001 | *** |
| Chlorogenic acid | 0.306 | 0.952 | 0.1428 | 0.335 |  |
| Vanillic acid | 0.911 | 0.411 | 0.9981 | 0.001 | *** |
| trans-Cinnamic acid | 0.998 | -0.069 | 0.9996 | 0.001 | *** |
| Syringic acid | 0.721 | -0.693 | 0.9428 | 0.001 | *** |
| Pinobanksin | -0.958 | 0.286 | 0.9998 | 0.001 | *** |
| Pinocembrin | -0.949 | 0.314 | 0.9998 | 0.001 | *** |
| Galangin | -0.913 | 0.408 | 0.9997 | 0.001 | *** |
| Chrysin | -0.897 | 0.441 | 0.9999 | 0.001 | *** |
| Kaempferol | 0.813 | 0.582 | 0.9985 | 0.001 | *** |
| Quercetin | 0.855 | 0.518 | 0.9992 | 0.001 | *** |
| Rutin | -0.995 | -0.099 | 0.2773 | 0.096 | . |
| Hyperoside | -0.190 | -0.982 | 0.9989 | 0.001 | *** |
| (±)-Naringenin | -0.495 | 0.869 | 0.9580 | 0.001 | *** |
| Myricetin | -0.171 | -0.985 | 0.9966 | 0.001 | *** |
| LD_50_ | -0.526 | 0.850 | 0.9999 | 0.001 | *** |
| Shannon | -0.154 | -0.988 | 0.9937 | 0.001 | *** |
| inv_Simpson | -0.217 | -0.976 | 0.9962 | 0.001 | *** |
| Richness | -0.727 | -0.686 | 0.9632 | 0.001 | *** |
| Chao1 | -0.969 | 0.249 | 0.5666 | 0.001 | *** |
| Ace | -0.988 | 0.158 | 0.5809 | 0.003 | ** |
| Pielou | 0.090 | -0.996 | 0.9958 | 0.001 | *** |

*p ≤ 0.05, **p ≤ 0.01, ***p ≤ 0.001

Reference:

Allegrini F, Olivieri AC (2020) Figures of Merit. In: Brown S, Tauler R, Walczak B (eds) Comprehensive Chemometrics: Chemical and Biochemical Data Analysis, vol 2. Elsevier Science, Boston, USA, pp 441-463. <https://doi.org/10.1016/b978-0-12-409547-2.14612-8>
